# Methodological advances enabled by the construction of a synthetic yeast genome

**DOI:** 10.1016/j.crmeth.2024.100761

**Published:** 2024-04-22

**Authors:** Daniel Schindler, Roy S.K. Walker, Yizhi Cai

**Affiliations:** 1 Max Planck Institute for Terrestrial Microbiology, 35043 Marburg, Germany; 2 Center for Synthetic Microbiology (SYNMIKRO), Philipps-University Marburg, 35032 Marburg, Germany; 3 School of Natural Sciences and ARC Centre of Excellence in Synthetic Biology, Macquarie University, Sydney, NSW 2109, Australia; 4 Manchester Institute of Biotechnology, University of Manchester, Manchester M1 7DN, UK

## Abstract

The international Synthetic Yeast Project (Sc2.0) aims to construct the first synthetic designer eukaryote genome. Over the past few years, the Sc2.0 consortium has achieved several significant milestones by synthesizing and characterizing all 16 nuclear chromosomes of the yeast *Saccharomyces cerevisiae*, as well as a 17^th^*de novo* neochromosome containing all nuclear tRNA genes. In this commentary, we discuss the recent technological advances achieved in this project and provide a perspective on how they will impact the emerging field of synthetic genomics in the future.

## Main text

### Introduction

With the advent of increased and economical DNA synthesis capacity, researchers are now able to consider designing and constructing DNA well beyond the scope and level of single-gene synthesis. This paradigm shift has enabled the construction of viral and bacterial genomes, as well as eukaryotic synthetic chromosomes, each of which represent significant milestones for the field of synthetic genomics.^1^^,^^2^ This commentary focuses on the Synthetic Yeast Project (Sc2.0), which is nearing completion following the synthesis and characterization of all 16 chromosomes plus the tRNA neochromosome. The majority of articles describing the synthesis and characterization of synthetic yeast chromosomes were published in two collections by Cell Press (https://www.cell.com/consortium/synthetic-yeast-genome) and Science (volume 335, issue 6329) in 2023 and 2017, respectively.

The success of Sc2.0 may lie in its organizational structure. Initially, the design of the synthetic chromosome sequences was centrally generated by using computer-aided design (CAD) with a clearly defined set of rules. The synthesis, construction, and characterization efforts were then distributed between several international laboratories, each of which led the construction and characterization of at least one synthetic chromosome and applied their own methodologies to do so. Notably, this franchise-like approach has greatly reduced the time taken to construct all 16 chromosomes and improved the efficiencies of scale for such a large project. Further advantages of this collaborative model include bringing together world-leading experts in their field in order to coordinate the detailed characterization and study of each synthetic chromosome.

The unique nature of synthetic eukaryotic genomics presents challenges but also an unprecedented opportunity to radically rethink the functional organization of the yeast genome. Thus, the design of the synthetic chromosomes was based on a few core design principles:^3^

• Integration of symmetric *loxP* sites three nucleotides downstream of nearly all non-essential genes or places where a genetic feature has been deleted
• Integration of PCRTags, short synonymously recoded regions in genes to allow to confirm presence of synthetic DNA by PCR
• Recoding of all TAG to TAA stop codons
• Removal of most introns except ribosomal protein introns
• Removal of transposable elements, subtelomeric repeats and dispersed genes with increased copy numbers
• Relocation of all tRNA genes to a dedicated neochromosome
• Minor sequence modifications to address the challenges of DNA synthesis (e.g., removal of restriction enzyme recognition sequences, modification of polynucleotide stretches).

Below, we outline some of the methodological advances and key technologies developed within Sc2.0. The individual steps represent important milestones for the field of synthetic genomics and are applicable beyond the field of yeast research. Furthermore, these synthetic yeast strains are increasingly being used for both basic and applied research. Some elements have already been transferred to engineer mice, for example, which will be highlighted in the concluding remarks.

### Synthesizing synthetic chromosomes by step-by-step replacement

**Figure 1:**
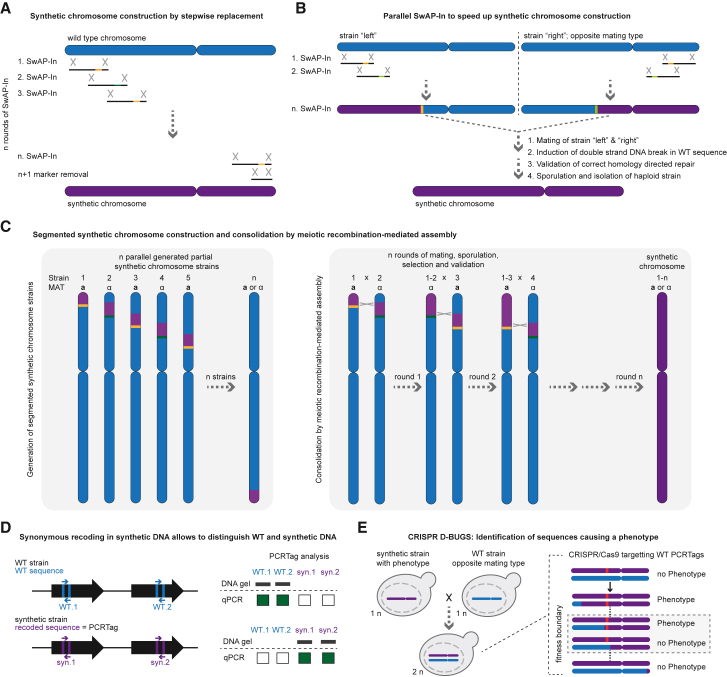
Construction of synthetic chromosomes and their systematic “debugging” (A) The stepwise replacement of WT DNA (blue) with its synthetic counterpart in multiple rounds of SwAP-In results in a fully synthetic chromosome (purple). Each round of SwAP-In is a round of transformation and selection, and between each round, the selection marker (orange or green) is swapped. In the final step, the selection marker is replaced with synthetic DNA by a counter selection approach, e.g., 5-FOA selection against *URA3*. X indicates homologous recombination sites. (B) SwAP-In can be parallelized, and a chromosome can be replaced stepwise from both ends. In this case, the WT DNA is replaced in parallel from both ends of the chromosome in two strains of opposite mating type. The two halves of the synthetic chromosomes are finally fused after mating by induction of double-strand breaks in the WT sequence. The endogenous homologous repair machinery will generate the complete synthetic chromosome by homologous recombination of the synthetic DNA, eliminating the selection markers in the same step. After validation of the synthetic chromosome, the strain must be sporulated and haploid candidates isolated for further validation and characterization. (C) Synthetic chromosome construction can be performed by segmenting the WT chromosome (blue) into n segments. The segments are replaced by synthetic DNA (purple) with alternating selection markers (orange or green) and overlapping sequence regions, resulting in n strains (left panel). In the n strain, the marker is removed to end up with a marker-free synthetic chromosome. The mating type must be altered to allow subsequent chromosome consolidation through successive rounds or mating and sporulation for the MRA (meiotic recombination-mediated assembly) technique, resulting in the final synthetic chromosome (right panel). (D) The wt and synthetic DNA sequences can be distinguished by PCRTags. PCRTags are short, synonymously encoded sequences within coding sequences that allow the generation of specific amplicons to validate the presence or absence of WT or synthetic DNA. PCRTag analysis can be performed by classical PCR followed by gel electrophoresis or by high-throughput endpoint genotyping qPCR. (E) CRISPR D-BUGS allows the identification of sequences responsible for a phenotype. The schematic shows the use of WT PCRTags to identify recessive defects. The method can also be reversed to identify dominant bugs by targeting the synthetic PCRTags. In both cases, the bug can be localized to the sequence region between two PCR tags where the phenotype disappears or appears (called the fitness boundary). The fitness boundary can then be carefully dissected and the phenotype causing sequence repaired. The red bar indicates the phenotype-causing sequence. Not shown is the integration of the *URA3* marker downstream of a telomere of the probed chromosome, which allows 5-FOA counter selection for the correct genotype.

One remarkable aspect of *S. cerevisiae* is its capacity for homologous recombination. By utilizing the endogenous homology-directed repair pathway in *S. cerevisiae*, which can efficiently join two DNA strands with little as 30 nucleotides of homology, endogenous DNA can be progressively replaced with chemically synthesized counterparts (Figure 1A). This method known as SwAP-In (switching auxotrophies progressively by integration) was developed to allow the progressive replacement of endogenous DNA by switching between two different selection markers with each round of DNA replacement.^3^^,^^4^ The approach was used to construct all 16 synthetic chromosomes for Sc2.0.

An inherent bottleneck is the stepwise nature of SwAP-In, with each round of integration taking a considerable amount of time (and even longer for larger synthetic chromosomes). This bottleneck may be alleviated by implementing an approach based on homologous recombination following double-strand breaks in a diploid strain. In this strategy, individual synthetic chromosome halves are constructed in parallel in two strains of opposite mating types (*MAT***a** and *MAT*α) (Figure 1B). For example, in each strain, replacement may be undertaken from the left telomere in one strain, and from the right telomere in the other. When both strains have reached a region of overlap following the introduction of synthetic DNA, the two strains are then mated, and a double-strand break is induced in the non-synthetic DNA sequence, allowing homologous recombination.^5^ The result of the double-strand break is the recombination of the two synthetic chromosome arms into a fully contiguous synthetic chromosome.

As chromosome length increases, synthetic chromosomes may be further divided into segments to reduce the time taken for their construction. Zhang and colleagues divided chromosome 12 (the largest yeast chromosome) and chromosome 4 (the largest yeast chromosome if the rDNA repeat is not counted) into 6 and 11 sections, respectively.^6^^,^^7^ The segmented strains were constructed in parallel and then combined using a method called MRA (meiotic recombination-mediated assembly). This methodology uses a hierarchical approach and multiple rounds of mating and sporulation to ultimately obtain a strain with a fully synthetic chromosome that does not rely on double-strand breaks (Figure 1C).

### Verification of successful replacement of endogenous DNA with its synthetic counterpart

Following each round of integration, it is necessary to validate the incorporation of synthetic DNA. This is done using a method called PCRTag analysis (Figure 1D). This technology uses synonymously recoded DNA sequences as a means to differentiate chemically synthesized DNA from its natural counterpart.^3^ By probing strains after each round of SwAP-In, PCRTag primer pairs may be used to rapidly validate the incorporation of synthetic DNA, thus speeding up synthetic chromosome construction and making it more economical by reducing the cost of whole-genome sequencing (WGS) as only PCRTag verified strains need to be validated by WGS.

The great number of PCRTags on a synthetic yeast chromosome renders traditional agarose electrophoresis cumbersome and expensive. By implementing state-of-the-art laboratory automation equipment and high-throughput qPCR, standard PCR reactions can be reduced to as low as 1 μL by simple endpoint genotyping qPCR in up to 1,536-well plates.^8^ This allows for rapid and easy PCRTag analysis at a fraction of the cost of standard PCR-based PCRTag analysis.

An advantage of the stepwise replacement of native DNA with its synthetic counterpart is the ability to quickly detect whether a particular DNA replacement causes a phenotype or fitness defect. Phenotypes caused by sequence changes in synthetic chromosomes are often referred to as “bugs” because of their analogy to software programming. While the identification and repair of bugs became a major challenge in the Sc2.0 project, it is a treasure trove for deeper understanding of biology.

### Identification and debugging of phenotypes caused by sequence alterations

Once the chromosomes have been replaced by their synthetic counterpart, the next step is sequence validation by next-generation sequencing (NGS). This process can identify minor sequence changes compared to the designed sequence as well as structural variations such as deletions, duplications, or inversions.^4^^,^^5^^,^^6^^,^^7^^,^^9^ For example, during the construction of synII, two duplicated regions were identified that could be eliminated using a strategy employing a *URA3* marker and the *I-Sce*I homing endonuclease recognition site.^5^ After genomic integration of the *URA3* marker and expression of *I-Sce*I, the induced double-strand break in the duplicated region is repaired by the endogenous homologous recombination machinery, eliminating the duplicated stretch of DNA.

Sequence alterations are easily detected by NGS, and their correction is straightforward using the molecular toolbox available for genetic engineering in yeast. However, when a phenotype is observed, it can be difficult to identify the causal mutation(s) responsible. As an example, during the characterization of synVII, it was observed that the transcript of *NSR1* was upregulated in RNA sequencing (RNA-seq) data, but Nsr1 protein abundance was decreased in proteome analysis.^10^ *NSR1* has multiple sequence changes according to the Sc2.0 design rules. Careful sequence dissection revealed that the loxPsym site integrated in the 5′ UTR was the cause of the observed phenotype, presumably causing structural changes in the *NSR1* mRNA, resulting in decreased translation efficiency.

Throughout the characterization process, omics technologies have been helpful in identifying sequences that cause phenotypes or are altered from the original sequence design. However, omics technologies are not always able to identify the individual sequence change that caused a given phenotype. In particular, during the process of synthetic chromosome consolidation into a single cell, small bugs can create synergistic defects when combined. Identifying the causal relationship between sequence change and phenotype will ultimately become more complex as the content of synthetic sequences increase in the consolidated genome. Ultimately, this may represent the final challenge of Sc2.0.

Recently, a strategy called CRISPR D-BUGS has been developed to rapidly identify the sequences that cause phenotypes (Figure 1E).^11^ CRISPR D-BUGS relies on exploiting the loss of heterozygosity in diploid cells by using sgRNAs targeting the PCRTag sequences to map the sequence causing the phenotype. PCRTag sequences are used because the wild-type (WT) and synthetic chromosomes differ at this position. CRISPR/Cas9-induced double-strand breaks allow the identification of the “fitness boundary” in the diploid background. The fitness boundary is the region where a defect appears or disappears, depending on whether it is a recessive or dominant defect. The strategy relies on the *URA3* marker integrated at one of the telomeres of either the WT (screen for recessive bugs) or synthetic chromosome (screen for dominant bugs) and 5-fluorotic acid (5-FOA) counter selection to obtain the correct karyotype after induction of Cas9 to probe for the presence of the phenotype. Figure 1D illustrates the mechanism of action to identify sequence regions containing recessive bugs; in this case, the *URA3* marker is integrated into the WT chromosome. Once the sequence has been identified, it is possible to dissect the sequence changes and potentially repair the fitness defect. CRISPR D-BUGS is a fast and reliable strategy that allows bug mapping in strains containing single or multiple synthetic chromosomes.

### Synthetic chromosome consolidation into a single cell

**Figure 2:**
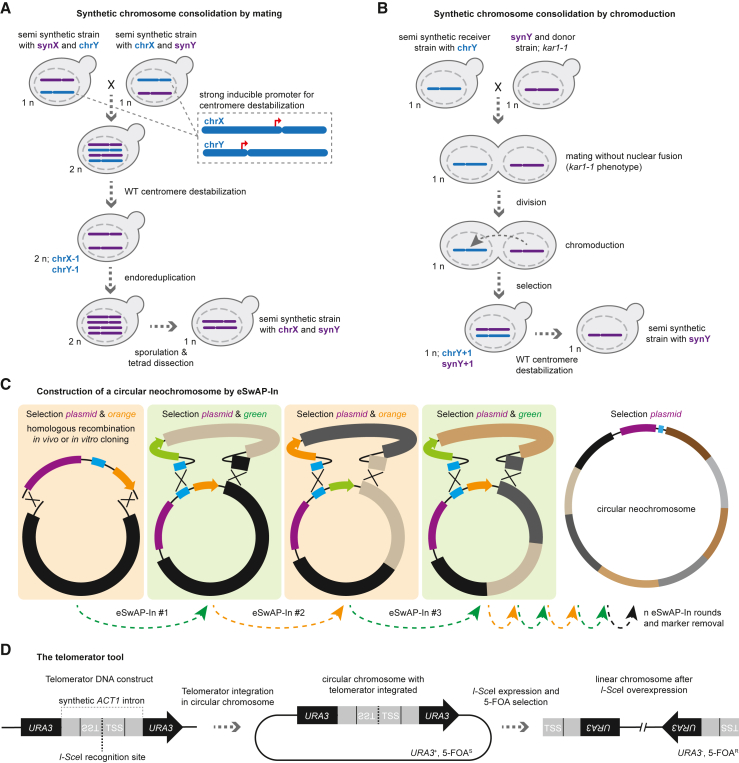
Synthetic chromosome consolidation and building a chromosome *de novo* (A) Multiple synthetic chromosomes can be consolidated into one cell by a process based on mating of semi-synthetic yeast strains and subsequent destabilization of the WT chromosomes. Destabilization of WT chromosomes is achieved by integrating a strong inducible promoter upstream of the centromere. Upon induction, transcription of the centromere region will prevent kinetochore formation and cause loss of the WT chromosome with the inactivated centromere. Endoreduplication may occur, resulting in two copies of the synthetic chromosome in the diploid cells. The diploid strain is then sporulated, and haploid isolates are obtained for further characterization steps and attempts to consolidate increasing numbers of synthetic chromosomes in a cell. (B) Chromoduction was developed to facilitate synthetic chromosome consolidation. The technique relies on the *kar1-1* allele, which allows cells of opposite mating types to mate, but their nuclei do not fuse. In some cases, however, individual chromosomes migrate from one nucleus to the other, allowing the selection of cells with a +1 aneuploidy for the intended synthetic chromosome to be transferred with the correct selection scheme. The WT chromosome can then be eliminated using the chromosome destabilization strategy (A). (C) Construction of circular neochromosomes can be achieved by repeated rounds of *in vivo* homologous recombination in yeast (eSwAP-In). The initial construct is generated by any suitable molecular cloning technique, followed by iterative rounds of DNA sequence integration using *in vivo* homologous recombination in yeast, facilitated by two homology arms containing the homologous sequences from the previous DNA segment and a universal homologous region (UHR; shown in blue). The sequential increase in size is illustrated by the colored blocks. Selection is achieved by recursive swapping of two markers, shown in orange and green, respectively. In a final step, the construction-based marker is removed, which, in a real case scenario, could be achieved via *URA3* and subsequent 5-FOA-mediated counter-selection. The purple bar indicates a plasmid originating region containing at least a centromere, an autonomously replicating sequence (ARS), and one selection marker (e.g., centromeric plasmids of the pRS41X series). (D) Sequence structure and use of the telomerator tool. The telomerator consists of the *URA3* marker with a synthetic intron based on the *ACT1* intron sequence. In addition, the intron contains two telomeric seed sequence (TSS) regions separated by an *I-Sce*I recognition site. The telomerator can be integrated into almost any position of a circular DNA construct. Upon induction of *I-Sce*I expression, the *I-Sce*I recognition site is cleaved, and the released TSSs can be recognized by telomerase and are extended and maintained as telomeres. Successful telomere formation would be indicated by a URA^−^ and 5-FOA^R^ phenotype. The telomerator can be used to convert any circular DNA construct into a linear chromosome as long as other sequence requirements, such as a centromere, are present.

All 16 synthetic chromosomes of Sc2.0 have been constructed in separate strains to parallelize their construction and characterization. The subsequent challenge is to consolidate all synthetic chromosomes into a single cell. For this purpose, a hierarchical mating procedure was developed that allows the stepwise combination of multiple synthetic chromosomes (Figure 2A).^3^ This strategy relies on the loss of WT chromosomes caused by introducing a strong inducible promoter adjacent to the WT centromere. After mating of the two semi-synthetic strains and induction of the promoter, the induced transcriptional machinery prevents the formation of the kinetochore, resulting in aneuploidy of the targeted chromosomes. The loss of WT chromosomes may be compensated by duplication of the synthetic chromosomes in a process called endoreduplication. The next step is sporulation of the respective strain followed by tetrad dissection to obtain haploid cells with the desired synthetic chromosomal complement. The resulting strains must be carefully tested for phenotypes and may need to undergo the debugging process described in the section above because additive fitness defects have been observed during chromosome consolidation.

The process of hierarchical mating-based chromosome consolidation is complex and relies on the construction of many strains. However, there are potential alternatives to this procedure. For instance, the *kar1-1* allele has been found to prevent nuclear fusion during mating, thus preventing the formation of diploid cells.^12^ However, in some cases, individual chromosomes are transferred between the nuclei while cells of opposite mating types are fused. This knowledge has been exploited for the consolidation of synthetic chromosomes, a process termed chromoduction (Figure 2B).^11^ By choosing the right selection conditions and subsequent centromere inactivation of wt chromosomes, this strategy allows rapid transfer of synthetic chromosomes into a recipient strain. It eliminates the need to construct several intermediate semi-synthetic strains, as was initially done during the synthetic chromosome consolidation process (Figure 2A). It also has the advantage that after successful chromoduction and karyotype validation, no additional sporulation and tetrad isolation is required. However, it is still mandatory to perform phenotyping throughout the process to identify potential errors caused by additive effects of multiple synthetic chromosomes.

### *De novo* chromosome design and construction

A new concept in synthetic genomics that has emerged from Sc2.0 is the concept of neochromosomes.^3^^,^^9^^,^^13^ Neochromosomes are supernumerary synthetic chromosomes with no natural counterpart. This concept enables the free design and construction of synthetic chromosomes that have user-defined characteristics and a limited number of prerequisites. These prerequisites are the elements required for chromosome maintenance that differ from the chosen host system, but they must fulfill the minimum requirements to allow replication and segregation of the chromosome in a cell-cycle-regulated manner. Thus, neochromosomes make it possible to study aspects of chromosome biology and to fundamentally test the elements required for chromosomal maintenance in a bottom-up manner.

To construct neochromosomes, extrachromosomal SwAP-In (eSAP-In) has been developed, which allows the successive construction of a supernumerary chromosome within the host cell (Figure 2C).^14^ This strategy initially relies on the stepwise assembly of a circular neochromosome, similar to the stepwise replacement of synthetic chromosomes, but each step adds new DNA-encoded sequence information instead of replacing existing DNA. Neochromosomes can also be constructed in a linear fashion, as shown in a recent work from the Dai lab.^13^ Construction of linear neochromosomes has certain advantages (for example, a lack of need to linearize after construction and a potential increase in stability), but the neochromosome cannot be selectively extracted for its subsequent transplantation due to topological differences between circular and linear chromosomes. The construction of a circular neochromosome has a major advantage in that it can be extracted from the host cell and transplanted into another cell.^9^ In subsequent steps, the circular chromosomes can be linearized using the telomerator tool developed by Leslie Mitchell and Jef Boeke (Figure 2D).^15^ The telomerator relies on a clever trick using the *URA3* marker intersected with a synthetic intron containing telomeric seed sequences (TSS) separated by the recognition site of the *I-Sce*I homing endonuclease. Upon *in vivo* expression of *I-Sce*I, the enzyme cleaves at a specific recognition site, thus releasing two telomere seed sequences that are acted upon by telomerase, thus forming functional telomeres. Selection for cells with linearized neochromosomes containing telomeres is performed by counter-selection using 5-FOA, as only cells with a disrupted *URA3* marker are able to grow. The advantage of this system is that a neochromosome can be linearized at almost any position, allowing the study of the biological effects of large changes in chromosome architecture.^9^^,^^15^

### Concluding remarks

This commentary may give the impression that Sc2.0 is mainly methodological and technological. However, this is not the case; each work generated by this project has significantly progressed the scientific research in addition to the numerous technological advances. Various synthetic chromosomes have already been used to improve the production of industrially relevant compounds (e.g., betulinic acid and lycopene) and to study biological phenomena using SCRaMbLE (synthetic chromosome rearrangement and modification by *loxPsym*-mediated evolution). SCRaMbLE relies on symmetric *loxP* sites that can rearrange DNA in the presence of the enzyme, Cre recombinase. Several SCRaMbLE applications are presented in the *Nature Communications* Yeast 2.0 collection (https://www.nature.com/collections/dhppvlvxxb). A highlight may be an initial study, in which the authors undertook the SCRaMbLEing of the circular right arm of synIX (synIXR).^16^ This study demonstrated for the first time that SCRaMbLE does indeed produce very high diversity from a single genotype. Of the 64 candidates selected, each contained unique structural rearrangements, demonstrating significant diversity from the 43 recombinase sites of synIXR. Notably, these isolates were later studied in detail using long-read direct RNA sequencing and provided new insights into how genome context can alter transcription and change transcript isoforms.^17^

The developments and concepts of Sc2.0 are already being used in other studies beyond yeast. For example, the methods developed for constructing large pieces of DNA are being used to engineer mice to understand the principles of mammalian *HoxA* cluster regulation by constructing 130 to 179 kb variations of the *HoxA* cluster and studying the results *in vivo*.^18^ The refactoring approach has also been transferred to the mouse model to study several disease-related loci, for example, by humanizing the viral receptor *ACE2* to model SARS-CoV-2 infection in mice.^19^ Notably, in this study, the loci were even “humanized,” potentially allowing future studies of human disease-causing factors in an *in vivo* system that represents a more human-like model of infection.

Thanks to Sc2.0 along with other synthetic genomics projects, much progress has been made in this emerging field. This field will continue to develop and provide new methodological advances to study “new biology.” The concept of neochromosomes and the systematic refactoring of large stretches of DNA will allow researchers to address current methodological challenges.

## Consortia

This work is part of the international Synthetic Yeast Genome Project (Sc2.0) consortium. The chromosome design and building consortium includes research groups worldwide: Boeke Lab at Johns Hopkins University and New York University (led chromosomes I, III, IV, VI, VIII, and IX); Chandrasegaran lab at Johns Hopkins (led chromosomes III and IX); Cai Lab at University of Edinburgh and University of Manchester (led chromosomes II and VII and the tRNA neochromosome); Yue Shen’s team at BGI-Research SHENZHEN (led chromosomes II, VII, and XIII); Y.J. Yuan’s team at Tianjin University (led chromosomes V and X); Dai Lab at Tsinghua University and Shenzhen Institute of Advanced Technology, CAS (led chromosome XII); Ellis Lab at Imperial College London (led chromosome XI); Sakkie Pretorius and Ian Paulsen’s team at Macquarie University (led chromosomes XIV and XVI); Matthew Wook Chang’s team at National University of Singapore (led chromosome XV); Bader and Boeke Labs at Johns Hopkins University (led design and workflow); and Build-A-Genome undergraduate teams at Johns Hopkins University and Loyola University Maryland (contributed to chromosomes I, III, IV, VIII, and IX). The Sc2.0 consortium includes numerous other participants who are acknowledged on the project website, www.syntheticyeast.org.
